# Feasibility, Reliability and Predictive Value Of In-Ambulance Heart Rate Variability Registration

**DOI:** 10.1371/journal.pone.0154834

**Published:** 2016-05-04

**Authors:** Laetitia Yperzeele, Robbert-Jan van Hooff, Ann De Smedt, Guy Nagels, Ives Hubloue, Jacques De Keyser, Raf Brouns

**Affiliations:** 1 Department of Neurology, Universitair Ziekenhuis Antwerpen, Edegem, Belgium; 2 Center for Neurosciences (C4N), Vrije Universiteit Brussel (VUB), Brussels, Belgium; 3 Department of Neurology, Universitair Ziekenhuis Brussel, Brussels, Belgium; 4 National MS Center, Melsbroek, Belgium; 5 Department of Emergency Medicine, Universitair Ziekenhuis Brussel, Brussels, Belgium; 6 Research Group on Emergency and Disaster Medicine Brussels, Vrije Universiteit Brussel (VUB), Brussels, Belgium; 7 Department of Neurology, University Medical Center Groningen, University of Groningen, Groningen, The Netherlands; University of Minnesota, UNITED STATES

## Abstract

**Background:**

Heart rate variability (HRV) is a parameter of autonomic nervous system function. A decrease of HRV has been associated with disease severity, risk of complications and prognosis in several conditions.

**Objective:**

We aim to investigate the feasibility and the reliability of in-ambulance HRV registration during emergency interventions, and to evaluate the association between prehospital HRV parameters, patient characteristics, vital parameters and short-term outcome.

**Methods:**

We conducted a prospective study using a non-invasive 2-lead ECG registration device in 55 patients transported by the paramedic intervention team of the Universitair Ziekenhuis Brussel. HRV assessment included time domain parameters, frequency domain parameters, nonlinear analysis, and time-frequency analysis. The correlation between HRV parameters and patient and outcome characteristics was analyzed and compared to controls.

**Results:**

Artifact and ectopic detection rates were higher in patients during ambulance transportation compared to controls in resting conditions, yet technical reasons precluding in-ambulance HRV analysis occurred in only 9.6% of cases. HRV acquisition was possible without safety issues or interference with routine emergency care. Reliability of the results was considered sufficient for Sample entropy (SampEn), good for the ratio of low frequency and high frequency components (LF/HF ratio) in the frequency and the time frequency domain, and excellent for the triangular interpolation of the NN interval histogram (TINN), and for the short-term scaling exponent of the detrended fluctuation analysis (DFA α1). HRV indices were significantly reduced inpatients with unfavorable outcome compared to patients with favorable outcome and controls. Multivariate analysis identified lower DFA α1 as an independent predictor of unfavorable outcome (OR, 0.155; 95% CI 0.024–0.966; *p* = 0.049).

**Conclusion:**

In-ambulance HRV registration is technically and operationally feasible and produces reliable results for parameters in the time, frequency, nonlinear and time frequency domain. Especially non-linear HRV analysis during emergency ambulance transportation may be a promising approach to predict patient outcome.

## Introduction

Heart rate variability (HRV) is the variation over time of the period between consecutive heartbeats (R-R interval). It reflects the net effect of sympathetic and parasympathetic inputs to the sinoatrial node. Decrease of HRV has been associated with worse clinical outcome in several medical conditions [1–9].

HRV assessment comprises analysis of R-R intervals obtained by ECG. A major advantage of this assessment is its reproducibility, but the recording duration varies significantly between studies. Generally, distinction is made between short-term registration (5–10 minutes) and long-term registration (at least 18 hours) [3].

Especially in the prehospital phase of emergency interventions, there is an unmet need for simple, inexpensive solutions to evaluate the patients’ autonomic response, which may reflect disease severity, anticipate complications and predict outcome. In this context, short-term registration of HRV using tiny, low-cost devices is the prime candidate to reliably measure autonomic dysfunction without disrupting the paramedics’ workflow during time-critical patient transportations. When including it in decision support systems, incorporated in in-ambulance telemedicine devices, it holds the promise to facilitate remote monitoring and medical decision making in the field [9–12]. Several recent studies evaluated ECG-derived HRV parameters for clinical decision support in the prehospital setting, but these mainly focused on the need for life saving interventions in trauma patients requiring helicopter transport (S1 Table) [7–9, 13–16].

This prospective, observational study explores the technical and operational feasibility and the reliability of short-term HRV registration during emergency ambulance transportation. Additionally it explores the association between prehospital HRV parameters, vital functions and patient outcome.

## Methods

### Study participants

All patients (≥ 18 years) transported during emergency missions by a Paramedical Intervention Team (PIT) of the Universitair Ziekenhuis Brussel between December 23^th^ 2013 and August 19^th^ 2014 were eligible for inclusion in the study. The PIT consists of two Emergency Medical Technicians (EMT) and one Critical Care Registered Nurse (CCRN). The study did not interfere with the dispatch or standing orders of the PIT. No interventions or medical decisions were based on HRV results. To avoid transportation and treatment delay in emergency settings, informed consent was obtained on opt-out basis followed by written informed consent from patients or legal representatives after the acute phase. The study protocol and consent process were approved by the ethics committee of the Universitair Ziekenhuis Brussel.

Data on patient characteristics (demographics, prehospital vital parameters, Glasgow Coma Scale (GCS) score, prehospital diagnosis, outcome and in-hospital mortality) were retrieved from the PIT reports and the medical hospital records. Unfavorable outcome was defined as need for admission to the intensive care unit (ICU), death during hospitalization, or need for prolonged hospitalization (> 30 days) after the emergency intervention.

A control population was included, consisting of age and gender matched volunteers. Data of the control population were obtained by history taking and from medical records, if available.

### HRV registration

HRV was registered using a non-invasive 2-lead ECG registration device (eMotion HRV, Motion Ltd., Kuopio, Finland), validated by the manufacturers for HRV measurement, especially for individuals without arrhythmia [17].

Compliant with safety regulations, all patients were secured on a stretcher in supine position during the ambulance transportation. The head of the stretcher was positioned according to the patient’s condition (preferably at 30 degrees). The ECG signal was acquired by applying two standard surface electrodes to the patients’ chest and one-button activation of the device. Prior to the start of the study, the CCRN nurses received training on the use of the device. The process was designed not to interfere with the paramedics’ workflow. Data was collected at a sampling rate of 1000 Hz/sec and digitally stored on the device. Additionally, HRV registration using the same device was performed in 50 volunteers in stationary, resting conditions.

### Preprocessing for HRV analysis

The ECG signal data was converted into text (.txt) files and a 5 minute segment was selected for retrospective analysis using the heart rate variability analysis software (HRVAS) [18]. Based on the timings recorded in the automated ambulance data log, we selected the first 5 minutes of registration recorded in a travelling ambulance for further analysis.

Beat annotations were obtained by automated analysis with manual review. The stored ECG data was screened for body movement, muscle and other artifacts and arrhythmias. Registrations were excluded if there was a technical problem with the recording or in case of inadequate length of registration. Registrations were considered unsuitable for further analysis if more than 25% of the segment consisted of ectopic beats and/or noise, or in case of non-sinus rhythm [16].

Preprocessing of the time series included ectopic interval detection using the median filter, ectopic interval correction using the cubic spline interpolation method and detrending using the wavelet packet technique with a cutoff frequency of 0.0391 Hz to remove very low frequency resulting from non-stationary signals [18].

### HRV parameters

HRV assessment included a myriad of parameters from the time domain, frequency domain, nonlinear analysis and time-frequency analysis. Time domain analysis uses parameters derived from direct measurements of intervals between adjacent QRS complexes resulting from sinus node depolarization (normal-to-normal R-R intervals, NN) or from the differences between these intervals [19]. Among the most frequently used are the basic statistical measures (e.g. standard deviation of all NN, the root-mean-square of differences of adjacent NN). Geometric methods may be more robust and include calculation of the HRV triangular index (TI), and the triangular interpolation of the NN interval histogram (TINN) [18, 19].

Power spectral analysis describes the periodic oscillations of the heart rate decomposed at different frequencies and amplitudes. The frequency domain is characterized by four major components: the high frequency (HF; 0.15–0.4 Hz), low frequency (LF; 0.04–0.15 Hz), very low frequency (<0.003–0.004 Hz), and ultra-low frequency (<0.003 Hz) components [20]. The ratio of LF and HF components (LF/HF ratio) is a surrogate parameter for the global sympathetic-parasympathetic equilibrium. A ratio >1 is an estimate of sympathetic dominance, while LF/HF<1 is associated with parasympathetic preeminence [18].

More recently, nonlinear techniques have been developed based on chaos theory and fractal geometry. They are generally considered to describe processes generated by biological systems in a more effective way. Frequently used methods include Poincaré plots, entropy based measures and detrended fluctuation analysis (DFA) [2, 3]. Sample entropy (SampEn) and the scaling exponent of the DFA (DFA α) are complex yet robust parameters for HRV analysis.

Time-frequency analysis allows simultaneous assessment of both time and frequency information via the windowed Fourier transform or the continuous wavelet transform. As the latter does not rely on the sinusoid waveform, it may provide access to information that may be obscured by Fourier analysis [18]. Especially the LF/HF ratio from the continuous wavelet transform scalogram, therefore, is an attractive parameter for HRV analysis.

For reasons of clarity, we focused our analysis on the most robust HRV parameters across the four domains: the TI and TINN for the time domain, the LF/HF ratio from autoregressive modeling for the frequency domain, Sample entropy (SampEn), DFA α, α1 and α2 for nonlinear analyses, and the LF/HF ratio from the continuous wavelet transform scalogram for the time-frequency domain. These parameters were selected based on literature findings on short-term HRV analysis in patients with acute pathology and in prehospital emergency conditions [2–9, 15, 16, 21, 22].

### Statistical analysis

Statistical analysis was performed using SPSS statistics version 22.0 (SPSS, Chicago, IL, USA). All parameters were checked for normality by visually inspecting the histograms and by considering the Shapiro-Wilk test. Parameters with normal distribution are presented as means (standard deviation, SD) and univariate analysis was performed with Pearson’s r or the t-test. Median (interquartile range, IQR), Spearman’s ρ and the Mann-Whitney U test were applied for not-normally distributed variables. Pearson’s χ^2^-test was used for categorical variables.

Reliability of in-ambulance HRV registration was assessed by split-half reliability testing using the Spearman-Brown correction with calculation of the half-test reliability coefficient (rhh) and Cronbach’s α. Absolute and relative reliability were assed using methods considered appropriate for short-term measurements of HRV [23]. Absolute reliability was assessed by the 95% limits of agreement with construction of Bland-Altman plots, in two steps as recommended.[24] First, the means of the differences between the two series of measurements were compared with zero, using a paired t-test. Then, the relative (positive or negative) differences within each pair of measurements were plotted against the mean of the pair, to verify the assumption that it did not vary in any systematic way over the range of measurements. Relative reliability was assessed by calculating the intraclass correlation coefficient (ICC), using the results of the average measures, reflecting the ratio of between-subject to the total variance. The data was considered sufficiently reproducible if the ICC was > 0.70, good if the ICC was > 0.80 and excellent if it was > 0.90 [23].

Univariate testing was performed to compare HRV parameters in patients and controls, and to identify associations between parameters reflecting HRV and patient demographics, vitals, prehospital diagnosis, prehospital treatment, and outcome parameters. Multivariate regression analysis by a backward conditional method was performed for HRV indices with a univariate *p*-value of <0.05. Patient demographics (age, gender) and prehospital vitals (systolic blood pressure (SBP), diastolic blood pressure (DBP), heart rate, blood oxygen saturation (spO^2^) and Glasgow Coma Scale score (GCS)) were added as covariates.

## Results

### Patient and control population

**Fig 1:**
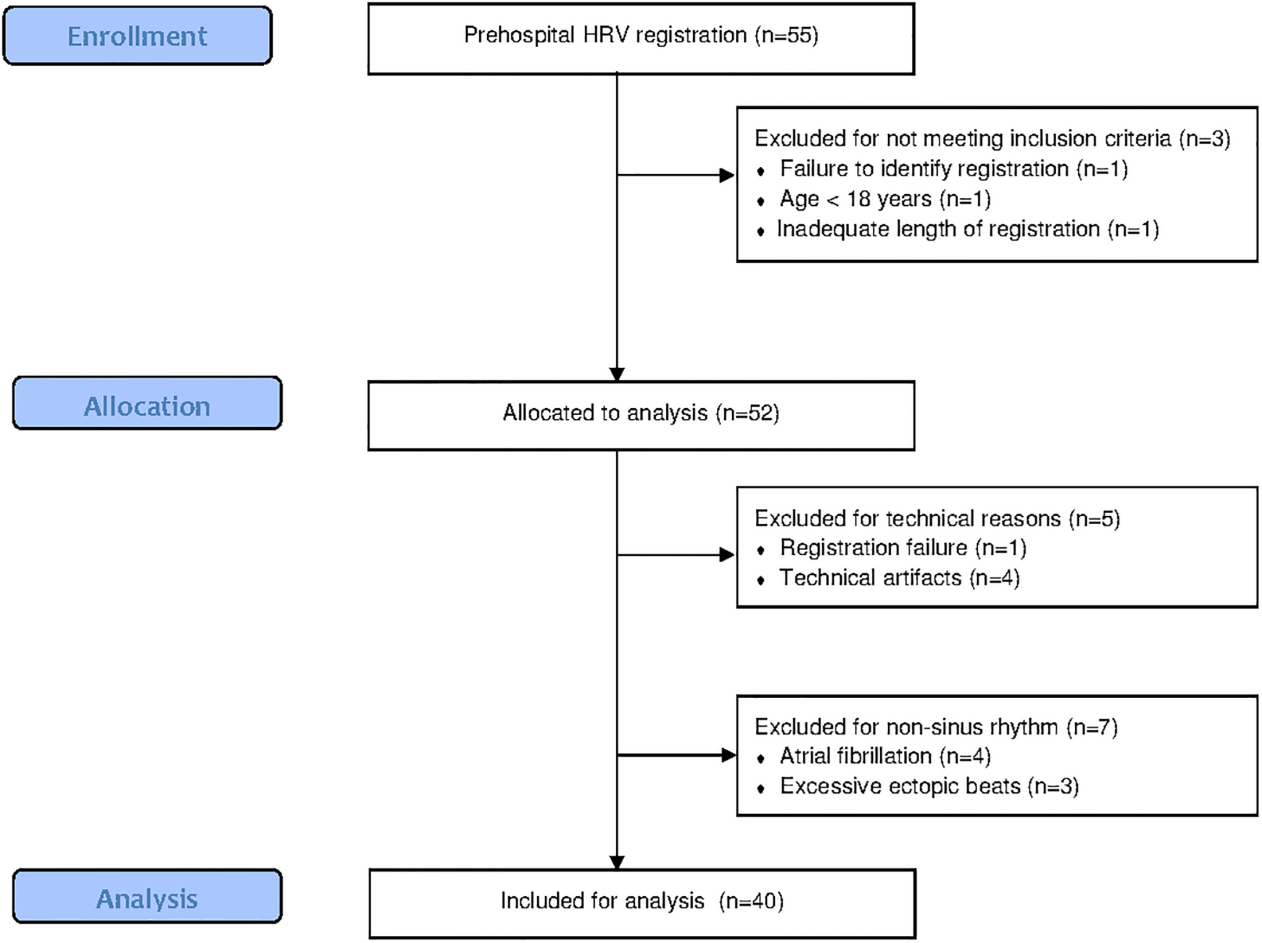
Flow diagram of HRV acquisition during ambulance transportation. HRV was registered in 50 controls. Three registrations were excluded from analysis: one because of technical reasons and 2 because of non-sinus rhythm. HRV parameters were analyzed in 47 controls, matched to the patient population for age and gender (S2 Table).

Prehospital HRV registration was performed in 55 patients of whom 52 met the inclusion criteria. Reasons for exclusion were: failure to identify the registration (n = 1), patient’s age <18 years (n = 1) and inadequate length of registration (n = 1). Additionally, registrations were excluded from analysis because of technical reasons (n = 5; 9.6%) or arrhythmia (n = 7; 13.5%) (Fig 1). HRV was analyzed in 40 patients, with a median age of 68.6 years (IQR; 59.4–82.3). Twenty one males (52.5%) and 19 females (47.5%) were included.

### Patient data and medical data

Vital parameters registered during transportation included SBP (n = 39; mean 158 mmHg; SD, 29), DBP (n = 39; median 90 mmHg; SD, 17), heart rate (n = 40; 94 bpm; SD, 21), spO^2^ (n = 40; median 97%; IQR, 95–98), glycaemia (n = 24; median 139 mg/dl; IQR, 102–192), and GCS (median 15; IQR, 14–15). Ten patients (25.0%) were prehospitally diagnosed with stroke, 5 with respiratory distress (12.5%), 3 with acute coronary syndrome (7.5%), 2 with severe trauma (5.0%) and 1 with intoxication (2.5%). Seven patients (17.5%) received prehospital medication, including morphinomimetics in 4 patients (10.0%), an anticholinergic or sympathicomimetic agent in 3 patients each (7.5%), antiaggregants in 2 patients (5.0%), and an anticoagulant in 1 patient (2.5%).

After treatment at the emergency department, 9 patients were discharged home (22.5%) and 30 patients required hospitalization (75.0%), of which 4 patients were admitted to the ICU (13.3%). The median hospitalization duration was 11 days (IQR, 6–17). Four patients died during hospitalization (10.0%). Twenty-seven patients were discharged home (67.5%), 6 to a rehabilitation facility (15.0%) and 2 to a nursery home (5.0%). In total, 15 patients (37.5%) had an unfavorable outcome at 30 days. Outcome data was missing in 1 patient.

### Transportation characteristics

Median ambulance transportation time was 6 minutes (IQR, 5–8 minutes). On average, 480 RR intervals were registered in the selected 5 minute fragment during ambulance transportation (SD, 104 intervals). The mean number of RR intervals was significantly lower in the control group (373 intervals; SD, 65 intervals; *p<*0.001). The median proportion of artifacts and ectopics was significantly higher and outliers were significantly more prevalent in the patient group compared to controls (1.8% vs. 0.63%; *p =* 0.008 and 9.5% vs. 2.0%; *p =* 0.003, respectively).

A subgroup analysis in 14 patients with a 5 minute ECG registration fragment available just before ambulance departure showed a similar number of RR intervals compared to their registration during transportation (488 ± 102 vs. 480 ± 104 RR intervals; *p =* 0.803), and therefore excluding a potential effect of transportation as a cause of tachycardia. There was no significant difference in artifacts and ectopics or outliers (*p =* 0.752 and 0.767, respectively), nor in the results for the key HRV parameters (data not shown) when compared to the recorded fragment prior to ambulance departure.

### Safety and acceptability

No adverse events or safety issues occurred during the study. No interference with the paramedics’ work-flow was reported. None of the patients refused participation to the study.

### Reliability

**Fig 2:**
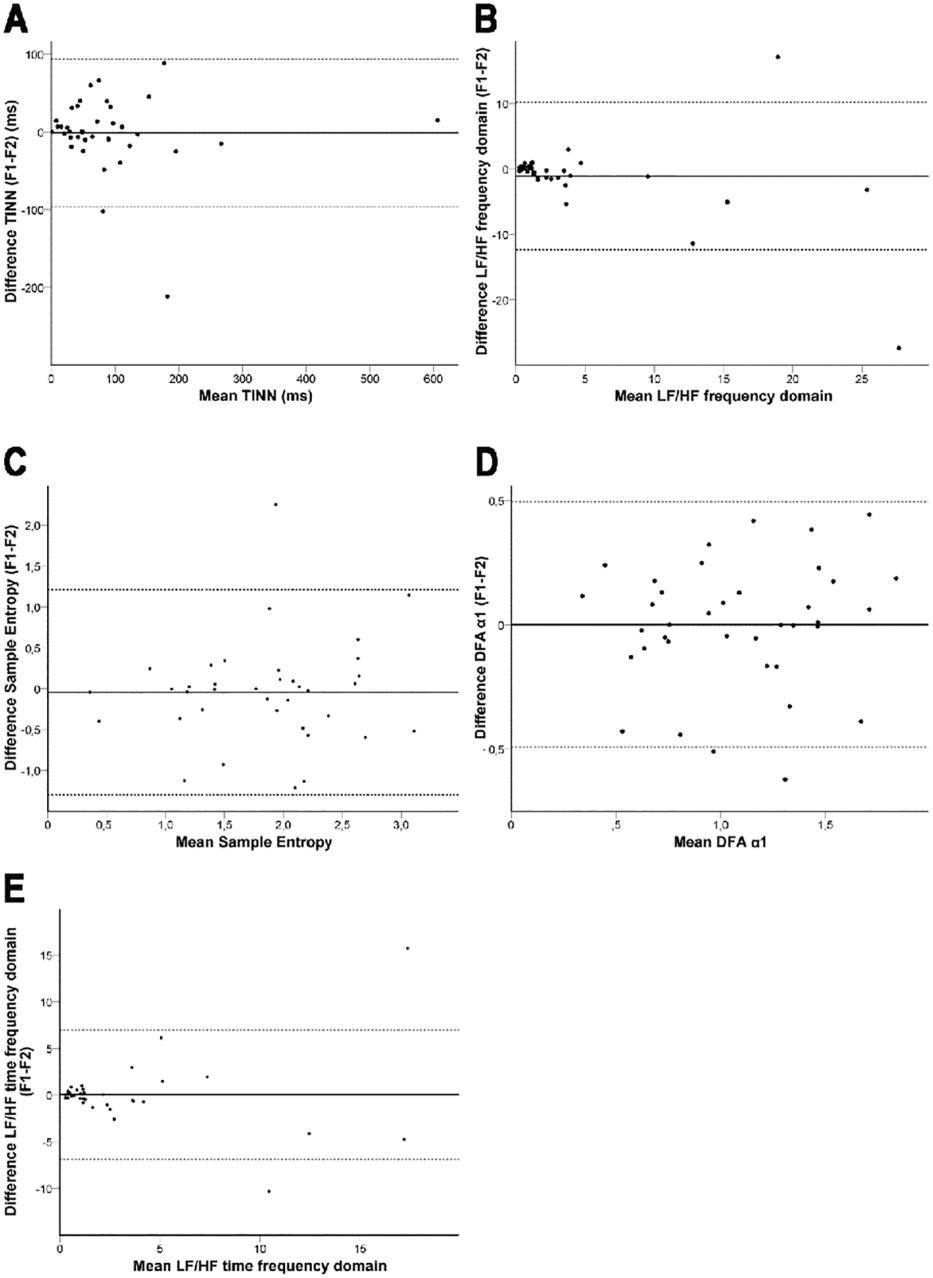
Bland-Altman plots with 95% limits of agreement for key HRV indices. Bland-Altman diagrams showing the mean differences ± 2 SD between measurement HRV F1 and F2.

There was no significant difference between the results of key HRV indices in the first and the second half (HRV F1: 0–150 sec; HRV F2: 151–300 sec) of the 5 min in-ambulance fragment (S3 Table). Evaluation of F1 and F2 using Pearson’s correlations, half-test reliability coefficients (*rhh*), Cronbach’s coefficient α, ICC, 95% limits of agreement (Table 1), and Bland-Altman plots (Fig 2) are concordant and strongly confirm the reliability of TINN, SampEn, DFA α1, and the LF/HF ratio in both the frequency domain and in the time frequency domain.

**Table 1:** Reliability of in-ambulance HRV parameters.

	Pearson’s *r*	*rhh*	Cronbach’s α	ICC	LOA
**Time domain**					
TINN (ms)	0.897	0.946	0.945	0.947	-96.09–93.81
**Frequency domain**					
LF/HF ratio	0.730	0.844	0.821	0.820	-12.35–10.22
**Nonlinear analyses**					
SampEn	0.628	0.772	0.770	0.774	-1,30–1,21
DFA α1	0.818	0.900	0.899	0.901	-0.49–0.49
**Time frequency domain**					
LF/HF ratio	0.719	0.837	0.835	0.839	-6.87–6.99

Abbreviations: DFA, detrended fluctuation analysis; HRV, heart rate variability; ICC, intraclass correlation coefficient; LF/HF ratio, low frequency/high frequency ratio; LOA, 95% limits of agreement; rhh, half-test reliability coefficient; SampEn, Sample Entropy; TINN, triangular interpolation of the NN interval histogram.

### HRV in patients and controls

HRV parameters in the control group were in line with literature findings and differed significantly from the patient population [25, 26]. When comparing the key HRV indices for each domain, TINN in the time domain, the LF/HF ratio in the frequency and the time frequency domain, and DFA α1 were significantly lower in the patient group compared to controls (Table 2).

**Table 2:** HRV parameters in patients and controls*

	Patient group *(n = 40)*	Control group *(n = 47)*	*P* value
**Time domain**			
mean HR°	97 (21)	75 (13)	*0*.*000*
TINN (ms)^#^	81.45 (IQR 54.40–133.97)	128.40 (88.60–179.40)	*0*.*002*
**Frequency domain**			
LF/HF ratio^#^	1.320 (0.609–3.692)	2.480 (1.469–4.138)	*0*.*041*
**Nonlinear analyses**			
SampEn^#^	1.839 (1.189–2.289)	1.762 (1.542–2.036)	*0*.*855*
DFA α1°	1.084 (SD 0.420)	1.262 (SD 0.279)	*0*.*025*
**Time frequency domain**			
LF/HF ratio^#^	1.117 (0.575–3.509)	2.155 (1.085–4.162)	*0*.*022*

* Data given as median (interquartile range) or mean (standard deviation);

^#^ Mann-Whitney U test;

° Student t test.

Abbreviations: DFA, detrended fluctuation analysis; HR, heart rate; HRV, heart rate variability; LF/HF ratio, low frequency/high frequency ratio; SampEn, Sample Entropy; TINN, triangular interpolation of the NN interval histogram.

### HRV and patient characteristics

Univariate analysis showed that age was inversely related with DFA α1 (ρ = -0.370; *p* = 0.019). There was no gender-based difference in the HRV parameters. Regarding the medical history, we found no association between TINN, LF/HF ratio, SampEn and DFA α1 and history of atrial fibrillation, stroke, arterial hypertension, diabetes mellitus, coronary artery disease, acute myocardial infarction, peripheral artery disease, dyslipidemia, obstructive sleep apnea, migraine, thyroid disease, obesity or epilepsy in this population. We found no correlation between HRV and the chronic use of medication, except for the LF/HF ratio in the frequency domain for patients taking sympathicomimetics (*p* = 0.023) and SampEn in patients taking anticoagulants (*p* = 0.029). For vital parameters, no correlation with SBP or DBP or with GCS score was found. There was a positive correlation between spO2 and the LF/HF ratio in the frequency and the time frequency domain (ρ = 0.376; *p* = 0.017 and ρ = 0.372; *p* = 0.018 respectively) and with DFA α1 (ρ = 0.422; *p* = 0.007). Glycaemia was inversely correlated with the LF/HF ratio in the frequency and the time frequency domain (ρ = -0.424; *p* = 0.039 and ρ = -0.421; *p* = 0.040 respectively). There was no association between HRV and prehospital administered medication, except for LF/HF ratio in the frequency domain (*p* = 0.042) and DFA α1 (*p* = 0.021) in patients who received morphinomometics.

### HRV and patient outcome

The risk of unfavorable outcome was associated with the LF/HF ratio in the frequency and the time frequency domain (*p* = 0.030 and *p* = 0.023 respectively), DFA α1 and SampEn (*p* = 0.038 and *p* = 0.021 respectively). The baseline characteristics of patients with favorable and those with unfavorable outcome are summarized in Table 3. We found no association between patient characteristics or prehospital vital parameters and unfavorable outcome at the level of *p*<0.10. When comparing HRV parameters in patients with favorable outcome to controls, no significant difference in HRV parameters was found.

**Table 3:** Baseline characteristics patient population*.

	Favorable outcome *(n = 24)*	Unfavorable outcome *(n = 15)*	*P* value
**Demographics**			
Male gender^§^	13 (54.2%)	19 (38.0%)	*0*.*748*
Age (years)^#^	64.9 (48.1–82.3)	72.4 (60.0–85.3)	*0*.*248*
**Vitals**			
GCS^#^	15 (14–15)	15 (12–15)	*0*.*744*
SBP (mmHg)°	157 (31)	159 (25)	*0*.*891*
DBP (mmHg)°	88 (19)	92 (16)	*0*.*586*
MAP (mmHg)°	111 (21)	114 (17)	*0*.*686*
HR (bpm)°	95 (18)	95 (26)	*0*.*990*
spO2 (%)^#^	97 (95–98)	96 (91–98)	*0*.*183*
Glycaemia (mg/dl) ^#^	147 (104–188)	120 (99–211)	*0*.*773*
**Medical history**^§^			
Arterial hypertension	9 (37.5%)	7 (46.7%)	*0*.*571*
Active smoking	2 (8.3%)	4 (26.7%)	*0*.*123*
Former smoking	2 (8.3%)	4 (26.7%)	*0*.*123*
Diabetes mellitus	11 (45.8%)	3 (20.0%)	*0*.*102*
Atrial fibrillation	3 (12.5%)	2 (13.3%)	*0*.*940*
Acute myocardial infarction	3 (12.5%)	1 (6.7%)	*0*.*559*
Coronary artery disease	2 (8.3%)	3 (20.0%)	*0*.*289*
Peripheral artery disease	1 (4.2%)	0 (0.0%)	*0*.*423*
Stroke	3 (12.5%)	0 (0.0%)	*0*.*154*
Dyslipidemia	7 (29.2%)	5 (33.3%)	*0*.*784*
Obstructive sleep apnea	0 (0.0%)	2 (13.3%)	*0*.*066*
Migraine	0 (0.0%)	1 (2.1%)	*1*.*000*
Thyroid disease	3 (12.5%)	2 (13.3%)	*0*.*940*
Obesity	0 (0.0%)	1 (6.7%)	*0*.*200*
Epilepsy	3 (12.5%)	0 (0.0%)	*0*.*154*
**Chronic medication**^§^			
Beta-blocker	2 (8.3%)	3 (20.0%)	*0*.*289*
Central agonist	1 (4.2%)	0 (0.0%)	*0*.*423*
Calcium channel blocker	4 (16.7%)	2 (13.3%)	*0*.*779*
ACE inhibitor	4 (16.7%)	1 (6.7%)	*0*.*363*
Angiotensin II receptor blocker	2 (8.3%)	2 (13.3%)	*0*.*617*
Other antihypertensive agent	4 (16.7%)	4 (26.7%)	*0*.*452*
Anti-arrhythmic agent	1 (4.2%)	1 (6.7%)	*0*.*731*
Sympathicomimetic	2 (8.3%)	3 (20.0%)	*0*.*289*
Statin	7 (29.2%)	4 (26.7%)	*0*.*866*
Anticoagulant	3 (12.5%)	0 (0.0%)	*0*.*154*
Antiaggregant	8 (33.3%)	5 (33.3%)	*1*.*000*
Thyroid hormone	2 (8.3%)	2 (13.3%)	*0*.*617*
Morfinomimetic drug	4 (16.7%)	3 (20.0%)	*0*.*792*
**Prehospital medication**^§^			
Morphinomimetic	3 (12.5%)	1 (6.7%)	*1*.*000*
Anticholinergic	1 (4.2%)	2 (13.3%)	*0*.*547*
Sympathicomimetic	1 (4.2%)	2 (13.3%)	*0*.*547*
Anticoagulant	0 (0.0%)	1 (6.7%)	*0*.*385*
Antiaggregant	1 (4.2%)	1 (6.7%)	*1*.*000*
**Key HRV parameters**			
Mean HR°	98 (18)	97 (25)	*0*.*865*
TINN^#^	80.85 (58.52–143.12)	81.30 (44.00–122.80)	*0*.*544*
FD LF/HF ratio^#^	1.578 (0.899–3.531)	0.711 (0.335–3.168)	*0*.*030*
SampEn^#^	1.403 (0.928–2.183)	2.053 (1.527–2.557)	*0*.*021*
DFA α1°	1.187 (0.396)	0.895 (0.409)	*0*.*033*
TF LF/HF ratio^#^	1.827 (0.950–4.069)	0.603 (0.385–3.771)	*0*.*022*

* Data given as number (percentage), mean (SD) or as median (interquartile range);

^§^ Fisher’s exact test;

^#^ Mann-Whitney U test;

° Student t test.

Abbreviations: DFA, detrended fluctuation analysis; FD LF/HF, low frequency/high frequency ratio in the frequency domain; HRV, heart rate variability; SampEn, Sample Entropy; TINN, triangular interpolation of the NN interval histogram; TF LF/HF, low frequency/high frequency ratio in the time domain.

The need for hospitalization was associated with SampEn (*p* = 0.004) and with DFA α1 (*p* = 0.039). In-hospital mortality was associated with SampEn (*p* = 0.026). There was no association between HRV parameters and the length of stay.

A multivariate stepwise logistic regression analysis was used to determine independent predictors of unfavorable outcome, using key HRV indices with a univariate *p* value of < 0.05 and including all parameters which are known during prehospital ambulance transportation (age, gender, SBP, DBP, HR, spO2 and GCS). In this model, a lower DFA α1 was independently associated with an unfavorable outcome (OR, 0.155; 95% CI 0.024–0.966; *p* = 0.049). SampEn was also independently correlated with unfavorable outcome, but did not reach the level of statistical significance (OR, 2.224; 95% CI 0.818–6.0488; *p* = 0.117).

## Discussion

This study is the first to evaluate the feasibility and the reliability of HRV registration during emergency ambulance transportation. The dataset size in our study is comparable to previously reported studies in emergency settings, which all used datasets of 800 beats or less. Cooke *et al*.[7, 8] studied 2 minute fragments and King *et al*.[13] evaluated 200 beat fragments. Registration of noise free datasets of sufficient length in field conditions may be challenging, which explains the high exclusion rates reported in studies assessing HRV during emergency helicopter transportation [8, 9]. In our study, HRV was successfully registered in 90.4% patients attempted, with acceptable amounts of technical artifacts. This demonstrates that in-ambulance registration of HRV is feasible. Registration in a traveling ambulance was as technically feasible as registration prior to ambulance departure.

After excluding patients with non-sinus rhythm, we successfully analyzed HRV parameters in 40 registrations (72.7%) and demonstrated the reliability of key HRV parameters in all four domains. Especially the TINN and DFA α1 proved to have excellent reliability in our population. In the past, it has been argued that the wide variation between measurements within the same individual decreases interpretability and utility of HRV, and low reproducibility of short-term HRV assessment has been described [14, 23]. The results of the present study demonstrate that the TINN, LF/HF ratio, SampEn and DFA α1 can reliably and reproducibly be measured in a 5 minute fragment obtained during emergency ambulance transportation.

Additionally, we demonstrate that reduced HRV during emergency ambulance transportation is predictive of poor patient outcome. Moreover, DFA α1 is identified a robust HRV parameter that is independently associated with outcome. These findings further substantiate the potential of HRV analysis as a new vital parameter with predictive value for short-term outcome in the emergency setting [7–9, 13–16] and opens up perspectives for in-ambulance HRV assessment as a prehospital triage tool. Potentially in combination with mobile telemedicine [12], wireless vital parameter monitoring may enable continuous HRV analysis in the near future, which would allow HRV indices to be implemented in predictive models or decision support strategies.

The major strength of this study is its prospective design, using a standardized and therefore highly reliable and reproducible methods for data acquisition and data processing. By consistently using the same registration device under the same conditions and processing data using automated beat annotation and artifact correction, any form of inter- or intrarater variability was excluded. Additionally, we focused on the HRV parameters known to have the highest reliability and strongest predictive value for short term HRV registration in our population. The use of a low-cost, user-friendly device for ECG registration, resulted in high operational feasibility.

The HRV acquisition duration was limited to 5 minutes, which may be seen as a drawback. Yet, consensus criteria on quantification of HRV are nonexistent and the median ambulance transportation in this study was only 6 minutes. The rather small sample size may be another limitation, but our study population is in line with previous literature reports [12]. Future research in larger study populations with specific medical conditions is required to more definitively establish the value of HRV as a predictive clinical tool and to develop threshold criteria for triage. Third, exclusion of patients with non-sinus rhythm may have introduced a selection bias and could limit generalization of the results. Registration of HRV in controls in resting conditions rather than during ambulance transportation is also a limitation, but this design was preferred as it may be challenging or undesirable to obtain emergency ambulance transportation in healthy individuals. We also acknowledge that the use of TINN as a time domain parameter has some limitations in short-term registrations. The absence of a relevant difference between the two outcome groups could indicate that this parameter is less robust than the other HRV parameters. Currently, the major limitation lays in the requirement to preprocess raw HRV data for analysis, which hampers real-time HRV analysis. Future developments should focus on the development of algorithms that allow real-time analysis of HRV to allow in-field risk-stratification.

## Conclusions

We demonstrate that real-time HRV acquisition during emergency ambulance transportation using a low cost ECG device produces sufficiently noise free data to allow HRV analysis, and we establish the reliability of in-ambulance HRV registration for parameters in the time, frequency, nonlinear and time frequency domains. Prehospital HRV is reduced in patients with unfavorable outcome when compared to patients with favorable outcome and controls. Evaluation of HRV using nonlinear parameters may be useful to predict short-term outcome in prehospital emergency conditions. However, before widespread application of HRV analysis can be adopted in the prehospital setting, real-time interpretation of short-term interval data must be made available.

## Supporting Information

S1 Table Literature overview of clinical decision support systems in the prehospital setting using ECG-derived HRV parameters. ApEn: approximate entropy; (C): number of controls; DFA: detrend fluctuation analysis; FD-L: fractal dimension by curve length; HFnu: HF power in normalized units; HF/LFnu: HF to LF ratio in normalized units; LFnu: LF power in normalized units; LSI: lifesaving interventions; N: number of patients; NN: Normal-to-normal heart beat interval; SDNN: Standard deviation of NN intervals.
(DOCX)

S2 Table Baseline characteristics of patients and controls. *Data given as number (percentage) or as median (interquartile range); ^§^Fisher’s exact test; ^#^Mann-Whitney U test.
(DOCX)

S3 Table HRV parameters in 2 separate fragments. *Data given as median (interquartile range) or mean (standard deviation); ^#^Mann-Whitney U test; °Student t test.
(DOCX)

## Abbreviations

DFA: detrended fluctuation analysis
HRV: heart rate variability
HRV F1: (0–150 seconds)
HRV F2: (151–300 seconds)
LF/HF ratio: low frequency/high frequency ratio
SampEn: Sample Entropy
TI: triangular index
TINN: triangular interpolation of the NN interval histogram
