# Supplementary material for: Feasibility, Reliability and Predictive Value Of In-Ambulance Heart Rate Variability Registration
Source: PLoS One. 2016 May 4;11(5):e0154834. doi: 10.1371/journal.pone.0154834 (PMC4856404; doi:10.1371/journal.pone.0154834)
Supplement: S1 Table — ApEn: approximate entropy; (C): number of controls; DFA: detrend fluctuation analysis; FD-L: fractal dimension by curve length; HFnu: HF power in normalized units; HF/LFnu: HF to LF ratio in normalized units; LFnu: LF power in normalized units; LSI: lifesaving interventions; N: number of patients; NN: Normal-to-normal heart beat interval; SDNN: Standard deviation of NN intervals. (DOCX) [file pone.0154834.s001.docx]

**Online supplement 1: Literature overview of clinical decision support systems in the prehospital setting using ECG-derived HRV parameters**

| **Author** | **Study design** | **Patient population** | **N (C)** | **HRV analysis** | **Duration** | **Conclusions** |
| --- | --- | --- | --- | --- | --- | --- |
| King et al.(9) | Prospective inclusion  Retrospective analysis | Helicopter transported trauma patients | 75 | Time domain | 200 beats | SDNN accurate predictor of base excess, major injury or LSI |
| Cooke et al.(10) | Retrospective analysis | Helicopter transported traumatic hemorrage patients without TBI | 15 (15) | Time domain  Frequency domain | 120 seconds | Lower LFnu, higher HFnu, higher HF/LFnu associated with increased mortality  No difference of time domain parameters in subjects who died compared to those who survived  Frequence domain parameters confounded by GCS |
| Cooke et al.(11) | Retrospective analysis | Helicopter transported trauma patients | 42 (42) | Time domain  Frequency domain | 120 seconds | Lower LFnu, higher HFnu, higher HF/LFnu associated with increased mortality |
| Batchinski et al.(5) | Retrospective analysis | Helicopter transported trauma patients | 20 (11) | Time domain  Frequency domain  Entropy domain | 800 beats | ApEn independent predictor of in-hospital mortality |
| Rickards et al.(12) | Retrospective analysis | Helicopter transported trauma patients | 127 (32) | Time domain  Frequency domain  Entropy domain | 800 beats | FD-L uniquely able to select patients needing LSI, but high false negative rate |
| Cancio et al.(13) | Retrospective analysis | Helicopter transported trauma patients | 138 (54) | Frequency domain  Entropy domain | 800 beats | SampEn and DFA independently associated with LSI |
| Ong et al.(14) | Prospective inclusion  Retrospective analysis | EMS transported patients | 45 | Time domain  Frequency domain | > 120 seconds | RMSSD, TINN and AR-HF correlated significantly with death  LFnu, HFnu, LF/HF, AR LF nu, AR HF nu, AR LF/HF correlated significantly with ICU admission  LFnu, HF nu, LF/HF, AR HFnu, AR LF/HF correlated significantly with hospital admission  NN50, pNN50, LFnu, AR LFnu correlated significantly with length of stay |

ApEn: approximate entropy; (C): number of controls; DFA: detrend fluctuation analysis; FD-L: fractal dimension by curve length; HFnu: HF power in normalized units; HF/LFnu: HF to LF ratio in normalized units; LFnu: LF power in normalized units; LSI: lifesaving interventions; N: number of patients; NN: Normal-to-normal heart beat interval; SDNN: Standard deviation of NN intervals
