# Supplementary material for: Feasibility, Reliability and Predictive Value Of In-Ambulance Heart Rate Variability Registration
Source: PLoS One. 2016 May 4;11(5):e0154834. doi: 10.1371/journal.pone.0154834 (PMC4856404; doi:10.1371/journal.pone.0154834)
Supplement: S2 Table — *Data given as number (percentage) or as median (interquartile range); §Fisher’s exact test; #Mann-Whitney U test. (DOCX) [file pone.0154834.s002.docx]

**Online Supplement 2: Baseline characteristics of patients and controls.***

|  | **Study group**  ***(n=40)*** | **Control group**  ***(n=47)*** | ***P* value** |
| --- | --- | --- | --- |
| **Demographics** |  | | |
| Male gender^§^ | 21 (52.5%) | 19 (38.0%) | *0.187* |
| Age (years)^#^ | 68.6 (59.4-82.3) | 72.9 (61.3-80.1) | *0.450* |
| **Medical history**^§^ |  |  |  |
| Arterial hypertension | 17 (42.5%) | 25 (53.2%) | *0.323* |
| Active smoking | 6 (15.0%) | 8 (17.0%) | *0.799* |
| Former smoking | 6 (15.0%) | 7 (14.9%) | *0.989* |
| Diabetes mellitus | 15 (37.5%) | 6 (12.8%) | *0.008* |
| Cardiac arrhythmia | 0 (0.0%) | 2 (4.3%) | *0.189* |
| Atrial fibrillation | 5 (12.5%) | 4 (8.5%) | *0.545* |
| Acute myocardial infarction | 5 (12.5%) | 2 (4.3%) | *0.161* |
| Coronary artery disease | 5 (12.5%) | 3 (6.4%) | *0.328* |
| Peripheral artery disease | 1 (2.5%) | 1 (2.1%) | *0.909* |
| Stroke | 3 (7.5%) | 1 (2.1%) | *0.236* |
| Dyslipidemia | 12 (30.0%) | 18 (38.3%) | *0.420* |
| Obstructive sleep apnea | 2 (5.0%) | 2 (4.3%) | *0.869* |
| Migraine | 0 (0.0%) | 1 (2.1%) | *0.356* |
| Thyroid disease | 5 (12.5%) | 5 (10.6%) | *0.787* |
| Obesity | 1 (2.5%) | 5 (10.6%) | *0.138* |
| Epilepsy | 3 (7.5%) | 2 (4.3%) | *0.519* |
| Parkinson | 0 (0.0%) | 1 (2.1%) | *0.356* |
| **Chronic medication**^§^ |  |  |  |
| Beta-blocker | 5 (12.5%) | 11 (26.0%) | *0.193* |
| Alpha-blocker | 0 (0.0%) | 1 (2.0%) | *0.356* |
| Central agonist | 1 (2.5%) | 0 (0.0%) | *0.278* |
| Calcium channel blocker | 6 (15.0%) | 6 (12.8%) | *0.765* |
| ACE inhibitor | 5 (12.5%) | 11 (23.4%) | *0.193* |
| Angiotensin II receptor blocker | 5 (12.5%) | 6 (12.8%) | *0.970* |
| Other antihypertensive agent | 9 (22.5%) | 8 (17.0%) | *0.523* |
| Anti-arrhythmic agent | 2 (5.0%) | 1 (2.1%) | *0.467* |
| Sympathicomimetic | 5 (12.5%) | 1 (2.1%) | *0.059* |
| Estrogen | 0 (0.0%) | 1 (2.1%) | *0.356* |
| Statin | 11 (27.5%) | 16 (34.0%) | *0.513* |
| Anticoagulant | 3 (7.5%) | 4 (8.5%) | *0.864* |
| Antiaggregant | 14 (35.0%) | 14 (29.8%) | *0.606* |
| Thyroid hormone | 4 (10.0%) | 3 (6.40%) | *0.539* |
| Morphinomimetic drug | 7 (17.5%) | 1 (2.1%) | *0.014* |

*Data given as number (percentage) or as median (interquartile range); ^§^Fisher’s exact test; ^#^Mann-Whitney U test.
