# Supplementary material for: Feasibility, Reliability and Predictive Value Of In-Ambulance Heart Rate Variability Registration
Source: PLoS One. 2016 May 4;11(5):e0154834. doi: 10.1371/journal.pone.0154834 (PMC4856404; doi:10.1371/journal.pone.0154834)
Supplement: S3 Table — *Data given as median (interquartile range) or mean (standard deviation); #Mann-Whitney U test; °Student t test. (DOCX) [file pone.0154834.s003.docx]

**Online Supplement 3: HRV parameters in 2 separate fragments.***

|  | **HRV F1 *(n=40)*** | **HRV F2 *(n=38)*** | ***P* value** |
| --- | --- | --- | --- |
| **Time domain** |  | | |
| TI° | 9.052 (SD 2.930) | 9.521 (SD 2.464) | *0.488* |
| TINN (ms) ^#^ | 63.50 (IQR 31.72-107.52) | 63.85 (27.80-128.55) | *0.869* |
| **Frequency domain** |  | | |
| LF/HF ratio^#^ | 1.273 (0.700-3.397) | 1.636 (0.484-4.296) | *0.869* |
| **Nonlinear analyses** |  | | |
| SampEn^#^ | 1.808 (1.213-2.371) | 1.940 (1.324-2.493) | *0.689* |
| DFA α° | 0.937 (SD 0.281) | 0.94066 (SD 0.264) | *0.956* |
| DFA α1° | 1.105 (SD 0.437) | 1.077 (SD 0.402) | *0.768* |
| DFA α2° | 0.912 (SD 0.343) | 0.903 (SD 0.286) | *0.900* |
| **Time frequency domain** |  | | |
| LF/HF ratio^#^ | 1.234 (0.660-3.711) | 1.636 (0.484-4.296) | *0.682* |

*Data given as median (interquartile range) or mean (standard deviation); ^#^Mann-Whitney U test; °Student t test.

Abbreviations: DFA, detrended fluctuation analysis; HRV, heart rate variability; HRV F1 (0-150 seconds); HRV F2 (151-300 seconds); LF/HF ratio, low frequency/high frequency ratio; SampEn, Sample Entropy; TI, triangular index; TINN, triangular interpolation of the NN interval histogram.
